# Clinical Overview of Luteal Deficiency in Dairy Cattle

**DOI:** 10.3390/ani12151871

**Published:** 2022-07-22

**Authors:** Fernando López-Gatius, Irina Garcia-Ispierto

**Affiliations:** 1 Transfer in Bovine Reproduction SLu, 22300 Barbastro, Spain; 2 Agrotecnio Centre, 25198 Lleida, Spain; irina.garcia@udl.cat; 3 Department of Animal Science, University of Lleida, 25198 Lleida, Spain

**Keywords:** additional corpus luteum, diagnostic tools, heat stress, high milk production, repeat breeding

## Abstract

**Simple Summary:**

Luteal deficiency is defined as reduced progesterone production by the corpus luteum, either in the amount or duration, or both. The clinical manifestations include primary infertility and pregnancy loss during the late embryonic/early fetal period (30–50 days post-AI). This work provides a clinical overview of the current understanding of luteal deficiency and its association with low fertility in dairy cows.

**Abstract:**

Luteal deficiency is defined as reduced progesterone (P4) steroidogenesis by the corpus luteum (CL), either in the amount or duration, or both. This work provides a clinical overview of the current understanding of luteal deficiency and its association with low fertility in dairy cows. Low plasma P4 concentrations during the luteal phase post-artificial insemination (AI) are associated with lower conception rates. Treatments post-AI with P4, gonadotropin-releasing hormone (GnRH) or human chorionic gonadotropin (hCG) improve fertility in some conditions. Sub-luteal function during the late embryonic period (at pregnancy diagnosis, i.e., 28–34 days post-AI), is just one factor among other factors associated with pregnancy loss. Treatment with P4 in cows with one CL favors pregnancy maintenance, while GnRH treatment does the same in cows carrying twins. The diagnosis of sub-luteal function can be made clinically on the basis of plasma or milk P4 concentrations. Automated in-line milk P4 analysis systems to diagnose luteal activity emerge as a very interesting tool in dairy herds. Monitoring plasma or milk P4 concentrations with the help of Doppler ultrasonography to assess the CL function would allow individualizing the luteal phase support.

## 1. Introduction

Luteal phase deficiency was defined in 1949 as reduced progesterone (P4) steroidogenesis by the human corpus luteum (CL), either in the amount or duration, or both [1]. The clinical manifestations of the defect in women include primary infertility and repeated first trimester abortions [2,3]. As a consequence, luteal phase support during the first six weeks of gestation is regarded as an essential condition for the success of assisted human reproduction procedures [4,5]. In cattle, although the concept of luteal deficiency has been scarcely developed [6,7,8], the clinical manifestations of a poor luteal function are similar to those in humans. Indeed, therapies applied in the early luteal phase post-artificial insemination (AI) or during the late-embryonic period improve the fertility in some cow subpopulations. This work provides a clinical overview of the current understanding of luteal deficiency and its association with low fertility in dairy cattle.

## 2. The Early Luteal Phase of Pregnancy

Low plasma P4 concentrations during the luteal phase post-AI have been extensively associated with lower conception rates [9,10]. This demands treatments post-AI with P4, gonadotropin-releasing hormone (GnRH) or human chorionic gonadotropin (hCG) to improve fertility in some herds. In effect, the results of recent meta-analyses, including data from a total of 59,584 cows, indicate that P4, GnRH or hCG treatment in the early luteal phase of pregnancy improves fertility, particularly in cows of lower fertility [11,12,13]. Certainly, poor luteal activity following ovulation may turn a cow into a repeat breeder [14,15]. In high-producing dairy herds, the incidence of repeat breeding can greatly exceed 20% [16,17].

## 3. The Late Embryonic Period

The period of gestation is divided into embryonic, from conception to the end of differentiation (approximately 45 days), and fetal, from completion of differentiation to parturition [18]. In high-producing dairy herds, pregnancy diagnosis is commonly performed in the late embryonic period, and up to 20% of pregnancies are lost within 30–50 days of gestation [19,20,21]. Beyond this time interval, the risk of losses is much lower. In a similar way to the luteal period post-AI, reduced plasma P4 concentrations at pregnancy diagnosis have often been associated with pregnancy loss [21,22]. Two facts reinforce this perception. First, the presence of additional CL (more CL than the number of embryos) has been strongly linked to pregnancy maintenance [20]. Second, P4 treatment at pregnancy diagnosis may reduce the incidence of pregnancy loss in single pregnancies [23,24,25]. However, in contrast to the early luteal phase of pregnancy, treatment with GnRH or hCG at pregnancy diagnosis has not been found to reduce pregnancy loss in studies, including all pregnant cows [26,27], and P4 treatment may increase the likelihood of pregnancy loss up to three times in cows with two or more CL when compared to GnRH treatment [25]. Due to its immunosuppressive role, too high levels of P4 probably do not favor cases of spontaneous twin reduction in which conceptus remnants may determine pregnancy loss. In contrast, GnRH treatment favors pregnancy maintenance and is linked to an increased twin reduction rate in cows carrying twins [25,28].

The CL regression causes embryonic death, or if vice versa, luteal regression is detected at least 3 days after the detection of the embryonic death [29]. Sub-luteal function is just one factor among other factors associated with pregnancy loss. This explains why post-AI treatment with P4, GnRH or hCG appears to have no effect on subsequent pregnancy loss [30]. Consequently, sub-luteal function in the luteal phase post-AI can be independent from luteal deficiency in the late embryonic period. If so, therapies should be established for each time point.

## 4. Diagnostic Tools for Luteal Deficiency

**Figure 1:**
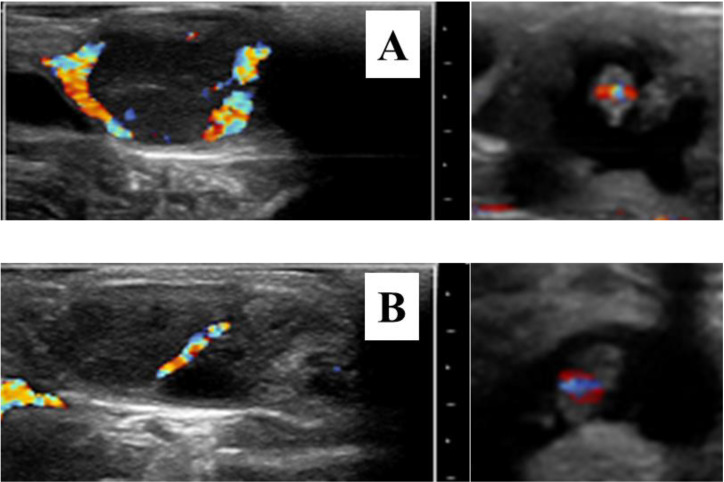
Color-flow sonograms of corpora lutea and their corresponding 30-day embryos showing normal (**A**) and low luteal vascular perfusion (**B**). The apparent luteal vascular decrease in (**B**) compared to that of (**A**) was associated with pregnancy loss 14 days later. All images were selected on the basis of maximal vascular perfusion from real-time video clips. Bar spacing: 10 mm.

Numerous mechanisms are involved in the formation and regulation of luteal structures [31,32]. So, we can expect that the function of the CL is influenced by many factors. For example, poor luteal activity has been associated with the intense metabolism and steroid hormone clearance of high milk production [33,34]. Heat stress is also a main factor impairing the CL function [35,36,37]. Luteal deficiency, therefore, is not due to a single etiology, and an etiologic diagnosis may be difficult or impossible to establish in routine clinical practice. Irrespective of its origin, the diagnosis of sub-luteal function can be performed clinically on the basis of plasma or milk P4 concentrations. Post-ovulatory increase in plasma P4 concentrations is strongly correlated with pregnancy success [38], while advanced (<7 days) or delayed (>11 days) onset of luteal activity post-AI has been associated with a decreased pregnancy rate (9.3 and 12.1%, respectively) when compared to the 7–11 days interval [39]. Eventually, low plasma P4 concentration during the late embryonic period has been related to pregnancy loss [40,41,42]. However, it is difficult to establish a reliable cut-off value for predicting pregnancy loss. It seems that high plasma P4 concentrations are a more useful predictor of pregnancy maintenance than low P4 concentrations are for predicting pregnancy loss [21]. In this context, sequential ovarian brightness (B)-mode ultrasonography plus plasma P4 measurements from AI to pregnancy diagnosis (day 32 post-AI) have proven to be useful tools in diagnosing luteal deficiency [8]. Or better, color-flow Doppler ultrasonography could be used to monitor appropriate luteal vascularization at the time of pregnancy diagnosis (Figure 1). Luteal blood flow, a strong indicator of luteal function, may provide additional information on luteal physiology compared to plasma P4 measurements alone [43,44,45]. Doppler ultrasonography is already used at the time of embryo transfer for selection of recipients [46,47,48]. However, the relationship of CL blood flow with pregnancy maintenance or pregnancy loss has been scarcely studied during the late embryonic period. A positive correlation between CL blood flow and plasma P4 concentrations has been reported in pregnant cows up to day 40 of gestation [49]. Normal and low luteal vascular perfusions shown in Figure 1 are just one example to be confirmed in extensive studies. Doppler blood flow studies should provide significant information about luteal deficiency during the late embryonic period and pregnancy loss and its treatments.

## 5. Clinical Perspectives

Pregnancy rate and pregnancy loss are two main reproductive parameters associated with luteal deficiency. Treatments during the early phase of pregnancy usually involve all inseminated cows [11,12,13]. Monitoring daily plasma or milk P4 concentrations during at least two or more cycles should allow individualizing luteal phase support. Cut-off values of P4 concentrations in different conditions are needed to confirm the effectiveness of adjusting treatments in cows with low P4 levels. For on-site assay of progesterone in milk, several validated enzyme immunoassay test kits have been developed, and some of them are commercially available [50,51,52], but they are too expensive to be included in daily screening programs. Even so, following a lengthy and laborious process [53], automated monitoring of P4 in the milking parlor is already possible with a new technology (in-line milk analysis system) [39,54,55,56]. Such systems may be an important tool for reproductive management in dairy herds [57].

## 6. The Situation in Other Mammalian Species

Luteal deficiency has been focused on in some studies on equine, porcine and dog, which may serve to illustrate major findings or divergences from the dairy cow model. Low levels of circulating P4 have been associated with pregnancy loss in the mare [58] and the bitch [59], and with pregnancy maintenance in the gilt [60]. Therefore, P4 therapy is recommended to reduce the incidence of pregnancy failure in mares and bitches of suspected sub-luteal function [59,60]. In contrast to the cow, controversial results are shown using Doppler ultrasonography to assess the CL function in the selection of equine embryo transfer recipients [61,62]. Turning the point to women, and as has been noted above, luteal deficiency has long been considered a main factor associated with low fertility [1,2,3,4,5]. It should be highlighted that, although the clinical manifestations of a poor luteal activity are similar for women and cows, the intensive protocols used in assisted human reproduction may have had a negative influence on subsequent CL function. This makes P4-based therapies particularly indispensable in women [63,64].

## 7. Concluding Remarks

In this age in which we are living of transition from clinics to reproductive genomics [65], the establishment of pregnancy following insemination remains the primary goal in most dairy systems [66]. At this juncture, clinical procedures to improve fertility in high-producing dairy herds leave much scope for improvement. In light of the information on sub-luteal function and its influence on dairy cow fertility, three important points stand out. First, luteal deficiency should not be considered in the follow-up work of low fertility alone. Second, treatment with P4, GnRH or hCG in the early luteal phase of pregnancy works well in sub-fertile cows. During the late embryonic period, treatment with P4 favors pregnancy in cows with one CL, while GnRH treatment increases the rate of pregnancy survival in twin pregnancies. Third, monitoring plasma or milk P4 concentrations with the help of Doppler ultrasonography to assess the CL function would allow individualizing the luteal phase support. Large controlled trials will be necessary to improve the efficiency of individualized treatments of luteal deficiency.

## Data Availability

All the data provided were extracted from the cited references.
